# The *Drosophila* aryl hydrocarbon receptor ortholog, *spineless*, modulates survival and reproduction

**DOI:** 10.1016/j.biochi.2026.02.011

**Published:** 2026-02-12

**Authors:** Shelton G. Swint, Shengshuai Shan, Jessica M. Hoffman

**Affiliations:** Department of Biological Sciences, Augusta University, 1120 15th St., Augusta, GA, 30912, USA

**Keywords:** *Drosophila*, Longevity, Aryl hydrocarbon receptor, Tryptophan metabolism

## Abstract

The aryl hydrocarbon receptor (AhR) is a highly conserved, ligand-activated transcription factor in mammals involved in multiple physiological processes, including development, xenobiotic detoxification, and potentially aging, and some of the most potent activators of AhR are tryptophan metabolites. AhR manipulation across species has been shown to have conflicting results on aging phenotypes that are often tissue specific. To expand our understanding of AhR and aging, we studied AhR affects survival and reproduction in *Drosophila melanogaster*, whose genome contains an ortholog of AhR, *spineless* (*ss*). Our findings indicate that ss-deficient flies have a shorter lifespan than wildtype flies but interestingly exhibit reduced mortality until approximately 40 days of age. Similarly, ss-deficient flies are more stress resistant than wildtype at young ages, but this reverses in later age. Negative lifespan-shortening effects of tryptophan metabolites were mitigated in ss-deficient flies, suggesting that the effects of these metabolites are ss-reliant, similar to AhR in mammals. Overall, our preliminary work demonstrates an evolutionarily conserved role for AhR in the aging process and increases our knowledge of the role of AhR/ss on aging phenotypes.

## 1. Introduction

**Fig. 1.**
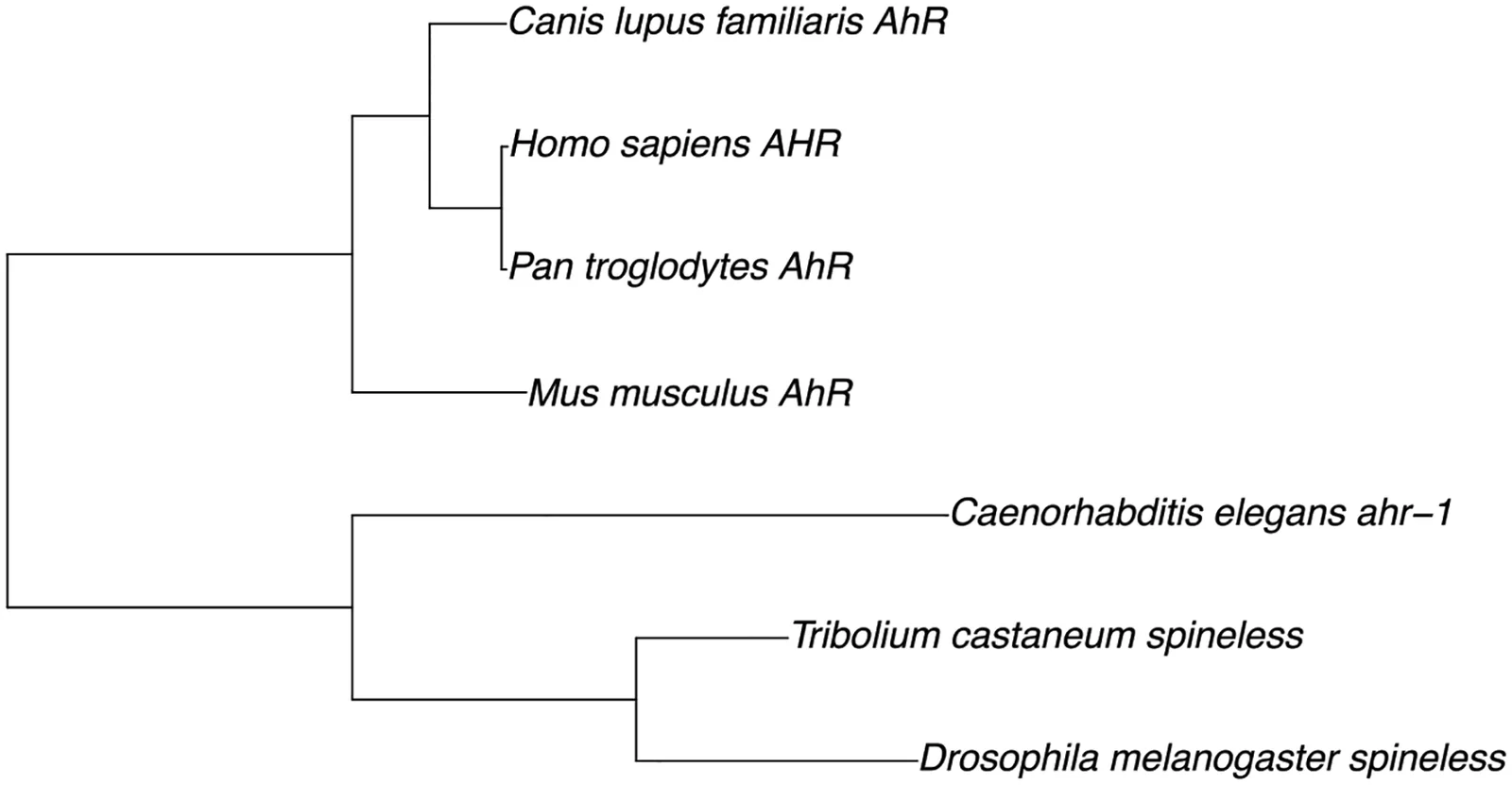
Phylogenetic tree of AhR orthologs.

Aging is a complex biological process influenced by various cellular mechanisms and metabolic pathways. As individuals age, normal physiological functions decline, leading to a higher risk of age-related diseases. We are just beginning to understand the mechanisms behind aging and how to develop interventions that can delay the aging process and extend healthspan. Interestingly, many age-associated pathways interact with the aryl hydrocarbon receptor (AhR) signaling pathway. AhR is a transcription factor that is highly conserved among mammals and has orthologs in other phyla (Fig. 1), including nematodes (ahr-1) and arthropods (spineless).

AhR is activated by both external xenobiotics and internal metabolites, especially those derived from tryptophan breakdown products. This activation increases cytochrome gene expression and subsequent production of reactive oxygen species (ROS) [1]. This process can cause excessive oxidative stress, leading to DNA damage and cell death, which negatively affects health and lifespan. In mammals, AhR is highly expressed in intestinal and lung epithelia, regulatory T cells, macrophages, and the liver [2]. Its other activities include developmental processes such as regulating immune, hematopoietic, and embryonic stem cell differentiation, as well as maintaining stem cell pluripotency [3]. AhR also promotes cellular senescence and apoptosis [4], contributing to inflammaging, a state of chronic, low-grade inflammation that worsens with age [5].

Because of its effects on multiple aging-related pathways, recent work has looked at the effects of AhR modulation on health and longevity. Studies using *Caenorhabditis elegans* with mutations in their AhR ortholog, ahr-1, have shown conflicting findings. Mutation of ahr-1 (ju145) in *C. elegans* resulted in improved healthspan and lifespan [6,7]. In contrast, Serna et al. observed an opposite effect from the same mutation in these worms, finding that this strain, along with another, ahr-1 (ia3), exhibited shorter lifespans compared to the N2 wildtype strain [8]. These studies differed in food sources and husbandry practices, and the latter study noted that these factors could have influenced the contradictory conclusions. Consequently, they repeated their experiments using the parameters set by Eckers et al. (2016) and reaffirmed their original finding that mutations in ahr-1 result in a shortened lifespan. Together, these studies suggest that the role of AhR in longevity may be more complex than previously thought, necessitating further investigation using other models, such as mice and flies.

In addition, Aarnio et al. (2010) found that worms with ahr-1 mutations exhibited reduced fecundity [9], similar to the reproductive deficits observed in AhR-null mice [10,11]. Studies on mice indicated that AhR-null individuals had significantly shorter lifespans [10,12], implying that AhR is essential for both reproductive success and longevity in mammals, much like in worms. Furthermore, human studies have demonstrated that AhR expression is higher in younger individuals (ages 30–50) and centenarians compared to septuagenarians [13], suggesting that AhR expression is crucial for longer, healthier lives. Collectively, findings from studies on worms, mice, and humans imply that AhR plays a complex role in the aging process across diverse phyla, where it is required for normal longevity but also promotes many late age negative effects like cellular senescence, and it has been suggested that AhR displays antagonistic pleiotropy [4]. So far, only one recent study has demonstrated the effects of a complete deletion of the *Drosophila* ortholog of AhR, spineless (*ss*) gene on fly longevity [14], which not surprisingly led to significantly reduced lifespan. We believe that further research on reduction of AhR (ss in flies), but not complete ablation, is necessary to better elucidate the complex role of AhR in the aging process and propose using *Drosophila melanogaster* to expand our collective knowledge on the role of AhR/spineless in aging phenotypes.

Spineless is involved in the developmental process of the legs, eyes, and neuronal dendrites in flies [15], as well as antennae and bristles [16]. ss cannot bind certain xenobiotic ligands that activate AhR in mammals, nor specific endogenous ligands like FICZ [17], and *Drosophila* do not suffer from dioxin toxicity due to incompatible binding to ss [15]. Its activity is ligand-independent, and ss constitutively heterodimerizes with tango (tgo) [15–17], the *Drosophila* ortholog of the mammalian AhR nuclear translocator (ARNT) protein. In this way, Céspedes et al. (2010) explain that while ss does not mediate dioxin responses in flies as AhR does in mammals, AhR and ss still serve very similar functions. For instance, flies expressing the murine AhR instead of their normal *ss* have normal leg morphology, with conclusions stating that AhR and ss functionally serve equivalent roles, except in mediating dioxin toxicity [15]. This could extend to other ligand-activated processes, in that ss may not respond and certainly does not require ligand activation, even though the ss PAS-B binding domain shares roughly 50% similarity with the mammalian ortholog on average [17–19].

The high similarity between binding domains suggests that flies could serve as a useful model for studying how AhR’s ligand-dependent modulation affects aging. Dai et al. (2022) demonstrate ss’s ability to bind to the AhR ligand α-naphthoflavone [17], so ss can bind some exogenous compounds that also bind AhR in mammals. However, there is still debate about whether other known AhR ligands, including tryptophan-derived metabolites, interact with ss. Tryptophan derived indoles and downstream metabolites are produced by bacteria and have recently been suggested to have longevity promoting effects [14,18]. In one study, the authors discovered beneficial effects from undefined indoles in *Drosophila*, such as improved climbing ability and increased stress resilience [19]; they also show that these effects are less pronounced in flies with an isogenic, loss-of-function mutation in *ss*. Similarly, Cao et al. (2025) claim that indole acetic acid (IAA) provides lifespan and healthspan extending effects with mechanisms potentially working through ss [14]. Lastly, our own recent studies show another indole derivative, indole-3 propionic acid (IPA), extends lifespan in flies [18], although the mechanism through which this occurs was not examined. Here, we examine the effects of ss on the lifespan and health of *Drosophila melanogaster*, and we explore potential interactions between tryptophan metabolites and ss to determine whether these interactions influence lifespan and health in flies.

## 2. Methods

### 2.1. Fly strains and husbandry

All stocks were raised on standard *Drosophila* media of cornmeal (6%), yeast (2.5%), sucrose (3.5%), dextrose (5.5%), agar (1%), and 5 mL/L propionic acid. Stock and experimental flies were kept in incubators at 25 °C and ~65% humidity on a 12-h light/dark cycle. A fly line with a hypomorphic mutation in *ss* was used throughout our experimentation: *w*^*1118*^*; PBac{WH]ss*^*f07846*^ (*ss-w*^*1118*^; Bloomington stock #19112). The wildtype line used was *w*^*1118*^ (Bloomington stock #3605). Homozygous stocks of *ss-w*^*1118*^ were used for experimentation. For all experiments, virgin flies were collected within 8 h of eclosion under CO_2_ anesthesia and sorted into sex specific vials of 20–25 flies, for a total of ~100 flies per experimental replicate.

### 2.2. Assays

For longevity analysis, flies were randomized onto appropriate treatments and transferred to fresh food three times a week until all flies were dead. Treatments of 5 g/L of L-Tryptophan (ThermoFisher Scientific #14059100) and 2.98 g/L of Tryptamine (ThermoFisher Scientific #186120500) were used in specified experiments. To determine neuromuscular function, climbing assays were performed at ~14, 20, 30, or 45 days of age following a previous protocol [20]. Vials had food dripped down their walls to provide extra grip since ss-deficient flies have abnormalities in their leg morphology. For oxidative stress assays, flies aged 10, 20, 30, or 40 days were placed on food containing 3% agar, 2% dextrose, 1% propionic acid, and 1.3% hydrogen peroxide. Deaths were tallied every 6 to 8 h. For a starvation assay, 14- and 40-day-old flies were placed on food containing 0.5% propionic acid and 1% agar. Vials were monitored for deaths every 6 to 8 h. For the body weight assay, 14-day-old flies were anesthetized on ice and weighed in an Eppendorf tube on a microbalance scale. The total weight was divided by the number of flies present in the vial. For the fecundity assay, five virgin females were mated with two virgin males for 48 h at 3 days of age. Vials were monitored for 14 days to record development stages: the day larvae started crawling, the day larvae pupated, and the day the first adult eclosed. Total progeny was determined by counting the number of live, adult offspring at 14 days.

### 2.3. Gene expression analysis

Gene expression levels were evaluated by quantitative reverse transcription-polymerase chain reaction (qRT-PCR), as described previously [21]. Two-week-old flies were flash-frozen in liquid nitrogen (~10 flies per tube) and stored at −80 °C. Subsequently, total RNA was extracted using a TRIzol/RNeasy (Plus Mini kit, Qiagen) hybrid extraction protocol. cDNA was produced using a high-capacity cDNA reverse transcription kit (4375575, Applied Biosystems) in 20 μl volume using 500 ng of total RNA, following the manufacturer’s protocol. cDNA was stored at −20 °C. Duplicate first strand cDNA aliquots for each sample served as templates for qPCR using SYBR Green PCR Master Mix (4309155, Applied Biosystems) on the QuantStudio 3 PCR System (A28137, Applied Biosystems). Forward and reverse primers for the *spineless* gene were CGAAGGCGACGCAACGG and GATGCCGCTTTGATGGATTGC, respectively. Forward and reverse primers for *vermillion*, the Drosophila ortholog of tryptophan 2,3-dioxygenase, were GGAAACGGAAACGATCACGAT and GGAGAGCATACACTGGGCA, respectively. Forward and reverse primers *Cyp6g1*, a Drosophila cytochrome, were TTGACCGAGGTCCTCTTTGTG and GGGCGGAATATAGGGTATGCC, respectively. *Drosophila* Actin 42A was used as an internal control with forward and reverse primers being GCGTCGGTCAATTCAATCTT and AAGCTGCAACCTCTTCGTCA, respectively. The high-efficiency assays enable relative quantitation to be performed using the comparative CT (ΔΔCT) method.

### 2.4. Phylogenetic analysis

Amino acid sequences of *AhR* from *Homo sapiens*, *Pan troglodytes*, *Mus musculus*, and *Canis lupus familiaris*, *ahr-1* from *C. elegans*, and *spineless* from *D. melanogaster* and *Tribolium castaneum* were obtained from NCBI. Sequences were aligned using NCBI’s COBALT Constraint-based Multiple Alignment Tool and downloaded as a NEXUS file. The phylogenetic tree was constructed in R using the msa [22], ape [23], and seqinr packages [24].

### 2.5. Statistical analysis

All statistical analyses were performed using GraphPad Prism or the R programming language [25]. Longevity, oxidative stress, and heat stress assays were analyzed using Cox proportional hazard models in the survival package [26], looking at the effects of sex, genotype, or treatment independently or interactions between treatment and genotype across sexes. Exponential hazard ratios (HR) and confidence intervals (CI) are reported in the figure legends. Mortality rates were calculated in Excel using formulae set by Promislow et al. and Ibrahim et al. [27,28]. The mortality rates were visualized using the same protocol and source code from Ibrahim et al. [26]. Climbing and reproductive assays were evaluated using either Wilcoxon tests or linear models for each sex, genotype, or treatment. RT-qPCR results were analyzed with t-tests. Results from all statistical analyses are shown in Supplementary Tables 1 and 2

## 3. Results

### 3.1. Normal levels of ss expression are required for longer lifespans, but increase early life mortality in Drosophila

**Fig. 2.**
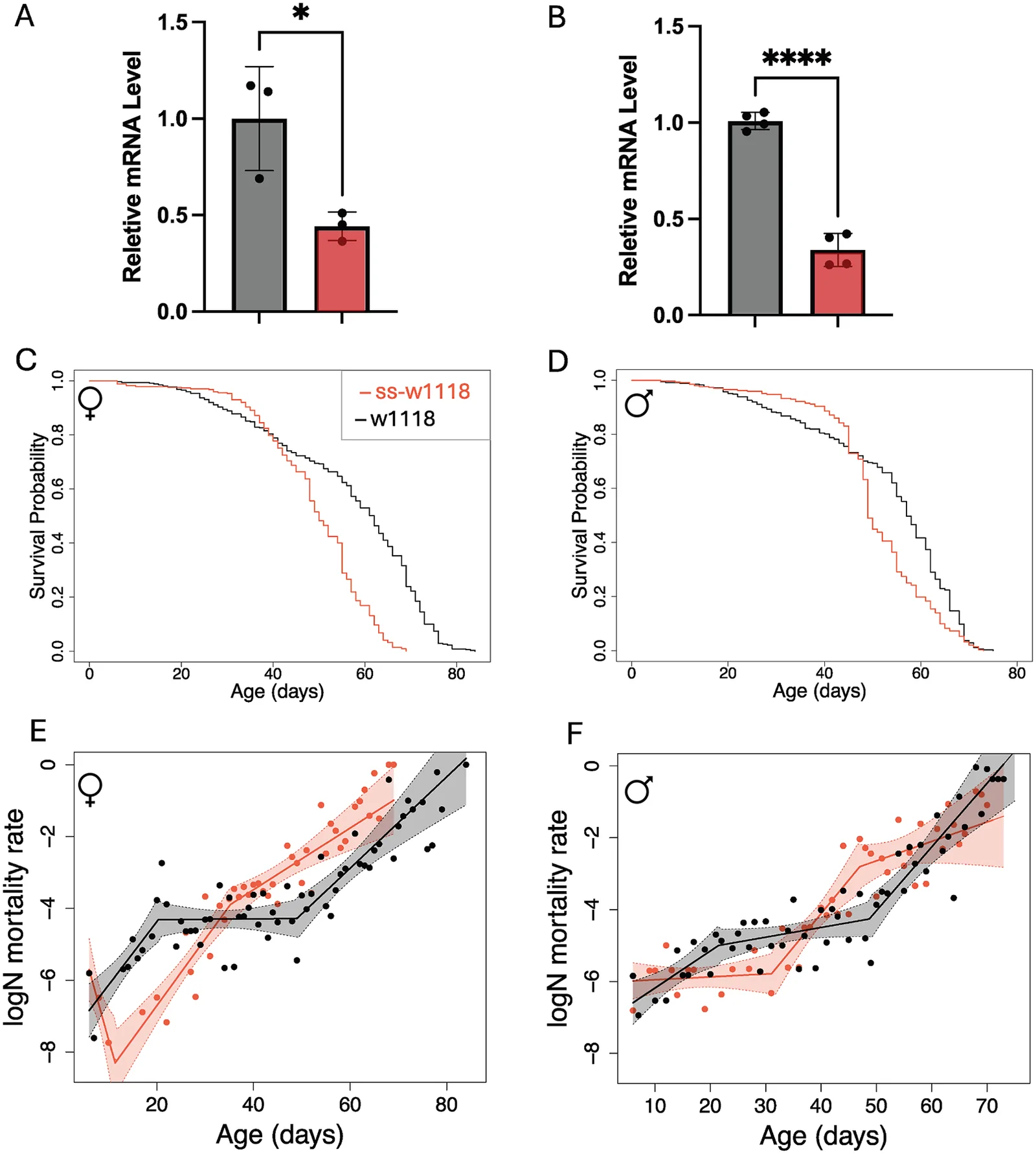
*ss*-mutants have reduced expression of *spineless* compared to wildtypes in (A) females (n = 120), and (B) males (n = 120). Survival analyses show *ss*-mutants have decreased longevity compared to wildtypes in (C) females (HR = 2.887 [95% CI, 2.418, 3.448], P = 1.20 × 10^−31^, n = 693) and (D) males (HR = 1.447 [95% CI, 1.244, 1.683], P = 1.70 × 10^−6^, n = 691) as measured across three replicates. Age-specific mortality rates are shown in E (females) and F (males). Wildtype flies are represented by black, and *ss*-mutant flies are represented by red. *p < 0.05, ****p < 0.0001.

*ss* expression was significantly reduced by more than 50% in both sexes (p < 0.05 for females and p < 0.0001 for males, Fig. 2A–B), indicating this mutation is a mild hypomorph. Longevity assays were performed over several replicates to measure lifespan across genotypes. ss mutant flies were shorter-lived compared to controls in females and males (p = 1.2 × 10^−31^ and p = 1.8 × 10^−6,^ respectively, Fig. 2C–D), but they exhibited improved survival in early life, as shown by a decrease in early life mortality in both sexes, more so in females (Fig. 2E–F).

### 3.2. Hypomorphic expression of ss results in improved resilience to stressors

**Fig. 3.**
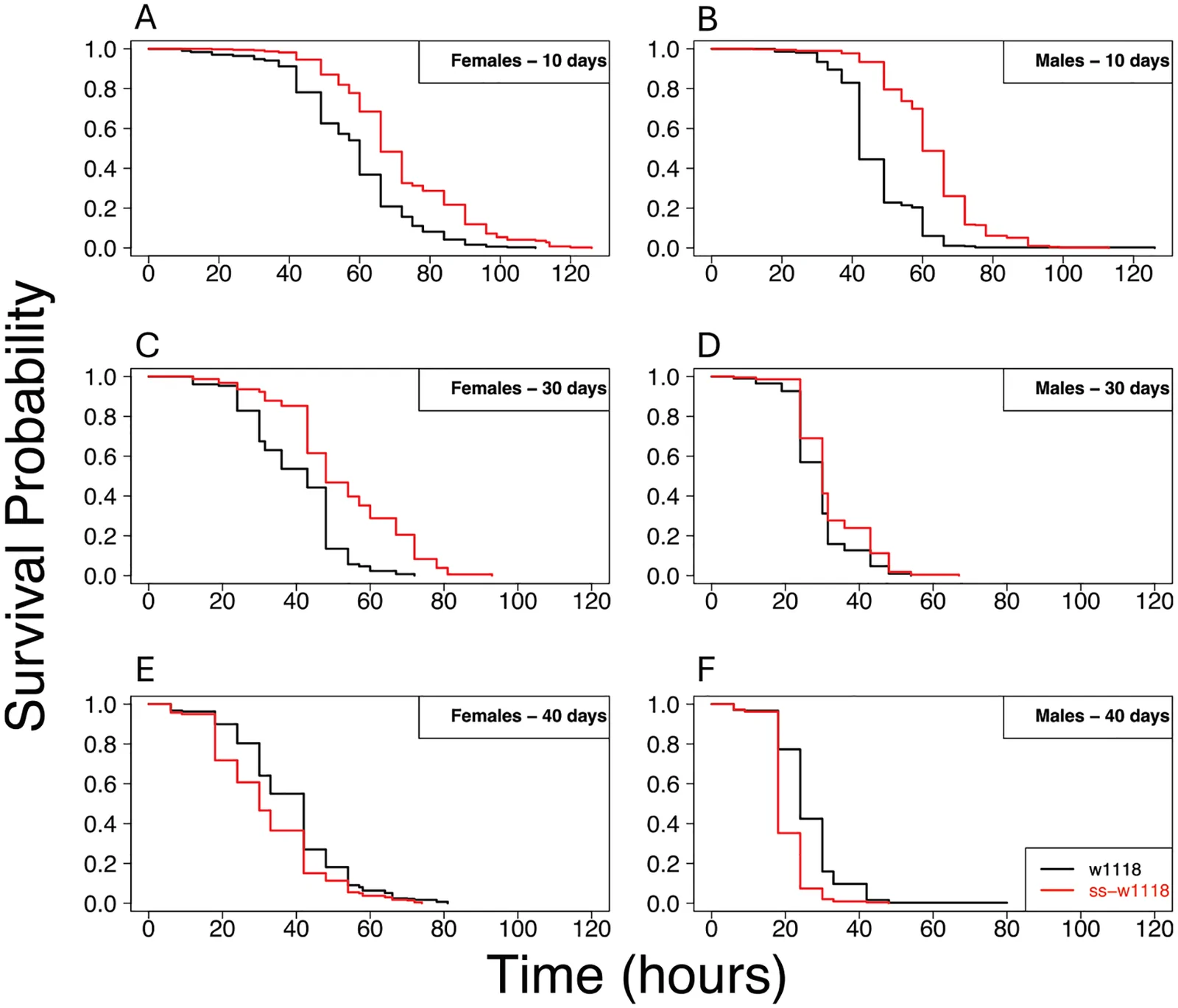
Oxidative stress assay survival curves of *ss*-mutants (red) and wildtypes (black), organized by sex (columns) and age at assay (rows). Two replicates were completed at each age. Sex and age of the flies tested are shown in the upper right corners. (A) Females at 10 days old (HR = 0.4756 [95% CI, 0.4068, 0.5559], P = 1.09 × 10^−20^, n = 694) and (B) males at 10 days old (HR = 0.2693 [95% CI, 0.2302, 0.3151], P = 2.60 × 10^−60^, n = 756). (C) Females at 30 days old (HR = 0.3698 [95% CI, 0.2993, 0.4570], P = 3.18 × 10^−20^, n = 540) and (D) males at 30 days old (HR = 0.7276 [95% CI, 0.6105, 0.8673], P = 3.85 × 10^−4^, n = 527). (E) Females at 40 days old (HR = 1.44 [95% CI, 1.253, 1.655], P = 2.94 × 10^−7^, n = 804) and (F) males at 40 days old (HR = 1.447 [95% CI, 1.244, 1.1.683], P = 1.70 × 10^−6^, n = 801).

**Fig. 4.**
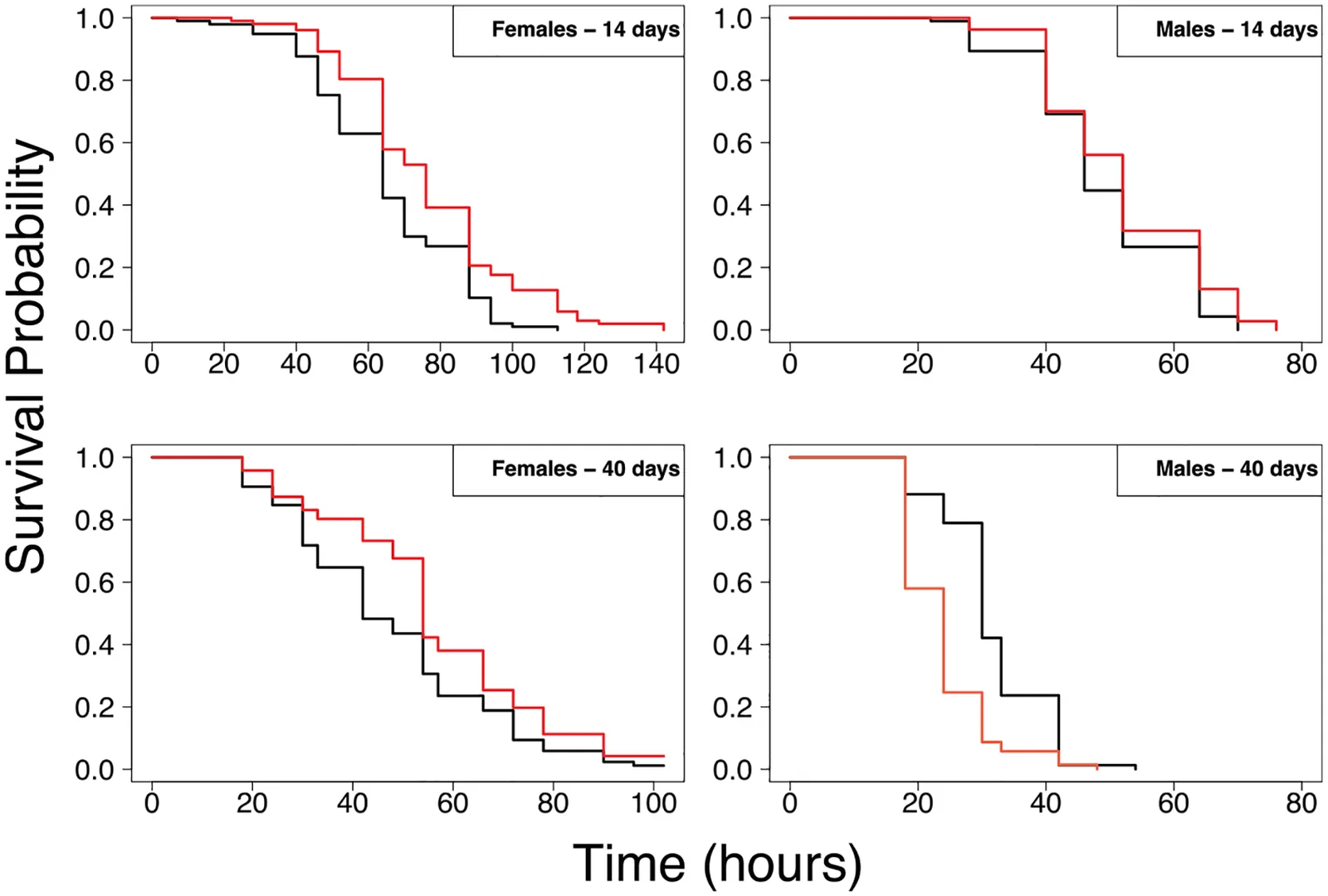
Starvation assay survival curves of *ss*-mutants (red) and wildtypes (black) organized by sex (columns) and age at assay (rows). One replicate was completed at both ages. Age of flies tested is shown in the upper right corners. (A) Females at 14 days old (HR = 0.5809 [95% CI, 0.4339, 0.7776], P = 2.62 × 10^−4^, n = 199) and (B) males at 14 days old (HR = 0.7458 [95% CI, 0.5619, 0.9898], P = 0.0423, n = 201). (C) Females at 40 days old (HR = 0.6949 [95% CI, 0.504, 0.9582], P = 0.0264, n = 156) and (D) males at 40 days old (HR = 2.437 [95% CI, 1.738, 3.417], P = 2.37 × 10^−7^, n = 145).

Previous research suggested that reduction in *ss* expression resulted in reduced activity of detoxifying genes [29], so we assessed the flies’ stress resistance using oxidative stress and starvation assays. At 10 and 30 days old, ss-deficient flies of both sexes were significantly more resilient to oxidative stress than wildtype flies (p < 1.1 × 10^−20^ for 10 days, p < 3.85 × 10^−4^ for 30 days, Fig. 3A–D), but less stress resistant at 40 days old (p < 3.0 × 10^−7^, Fig. 3E–F). Similarly, at a young age, female (p = 2.62 × 10^−4^, Fig. 4A) and male (p = 0.0423, Fig. 4B) ss-deficient flies exhibited improved starvation resistance. Interestingly, at 40 days old, ss-deficient females were still more resistant to starvation stress (p = 0.026, Fig. 4C), while ss-deficient males were significantly less resistant (p = 2.37 × 10^−7^, Fig. 4D).

### 3.3. ss is necessary for higher fecundity and faster development speed

**Fig. 5.**
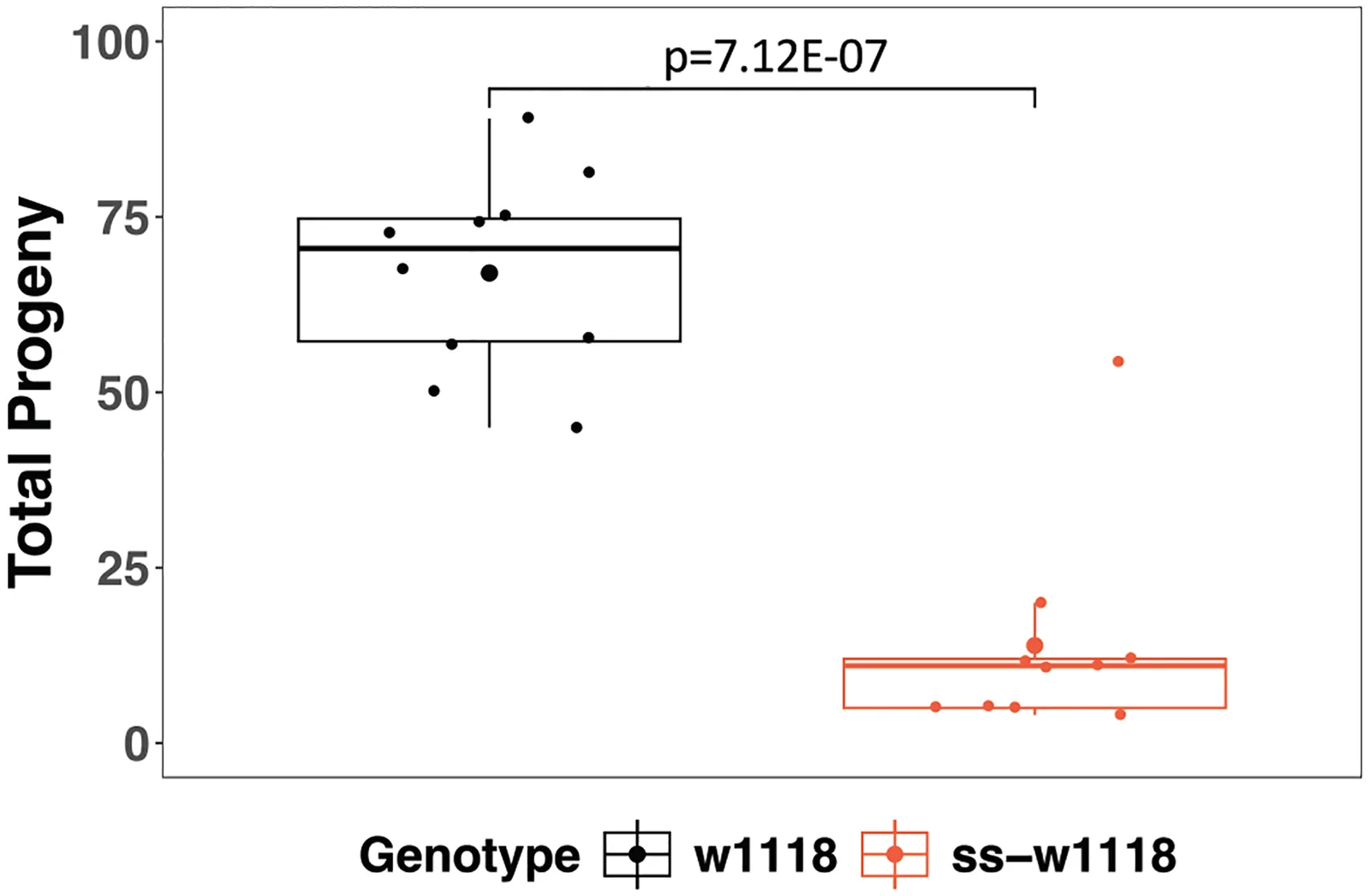
Reproductive output, as measured by total progeny produced by wildtype (black) and ss-deficient (red) flies, is dramatically reduced in ss-deficient flies (p = 7.12 × 10^−7^, n = 20 across two replicates).

To compare AhR’s effects on reproduction across species, we performed fecundity assays to measure reproductive output (total number of adult progeny produced) and the time required for developmental processes. Reproductive output was significantly lower in ss-deficient flies as compared to wildtype, as measured by the number of progeny (p = 7.12 × 10^−7^, Fig. 5). Development time, determined by the number of days to the first larvae crawling along the vial walls, pupation, and eclosion, was also significantly delayed in ss-deficient flies (p < 1.7 × 10^−5^, Fig. S1A–C).

### 3.4. Neuromuscular function appears to be disrupted in ss mutant flies

Decreases in neuromuscular function have been correlated with the aging phenotype. Climbing assays were employed to measure neuromuscular function in flies with normal expression of *ss* and *ss*-mutant flies. At all ages, female *ss*-hypomorphic flies exhibited weaker neuromuscular function than wildtype (p < 0.043, Fig. S2A). Male *ss*-hypomorph flies displayed a similar climbing ability to wildtype flies until 45 days of age, at which point they also experienced a significant reduction in neuromuscular function (p = 0.0357, Fig. S2A). Body weight assays showed that *ss*-hypomorph female flies weighed more than wildtype female flies (p = 5.41 × 10^−5^, Fig. S2B) with no difference in males.

### 3.5. Lower expression of ss correlates with reduced effects from high doses of tryptophan and tryptamine

**Fig. 6.**
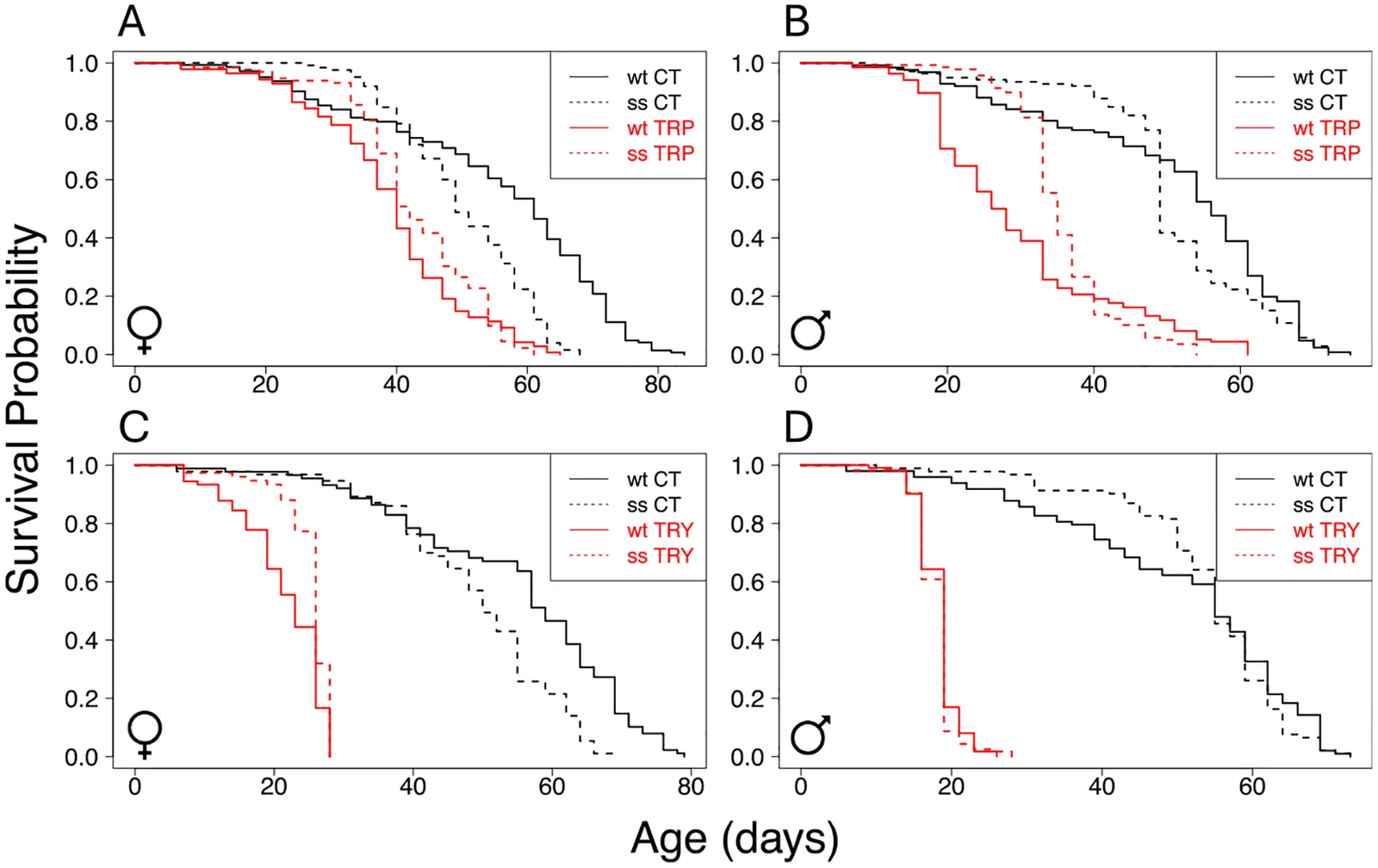
Survival curves of *ss*-mutant and wildtype treated with 5 g/L L-Tryptophan in (A) females (treatment with TRP HR = 5.810 [95% CI, 4.370, 7.723], P = 9.24 × 10^−34^; treatment-by-genotype interaction HR = 0.3315 [95% CI, 0.2304, 0.4769], P = 2.65 × 10^−9^, n = 542) and (B) males (treatment with TRP HR = 5.482 [95% CI, 4.218, 7.126], P = 4.72 × 10^−37^; treatment-by-genotype interaction HR = 0.7545 [95% CI, 0.5361, 1.062], P = 0.1060, n = 540). Survival curves of *ss*-mutants and wildtype treated with 2.98 g/L Tryptamine in (C) females (treatment with TRY HR = 138.81 [95% CI, 67.795, 284.21], P = 1.74 × 10^−41^; treatment-by-genotype interaction HR = 0.2713 [95% CI, 0.1730, 0.4256], P = 1.34 × 10^−8^, n = 346) and (D) males (treatment with TRY HR = 65.81 [95% CI, 34.979, 121.14], P = 1.18 × 10^−39^; treatment-by-genotype interaction HR = 1.057 [95% CI, 0.7161, 1.559], P = 0.781, n = 417). The data shown here are from one replicate each.

We were then interested in whether ss interacted with tryptophan metabolites, as established in mammals. We found that all groups had shorter lifespans when treated with high-dose tryptophan (p < 1.5 × 10^−22^, Fig. 6A–B), but a significant genotype-by-treatment interaction was observed in females, where the negative effects of tryptophan were more pronounced in wildtypes (p = 2.65 × 10^−9^, Fig. 6A–Table S1). A similar trend was observed in male tryptophan-treated flies, although no statistical significance was reached. (Fig. 6B). Similarly, tryptamine-supplemented flies lived significantly shorter than control flies in all groups (p < 1.2 × 10^−39^, Fig. 6C–D), but a treatment-by-genotype effect was observed in females, where *ss* females experienced a lesser impact from tryptophan (p = 1.34 × 10^−8^, Fig. 6C). Tryptophan treatment increased later-age climbing ability in wildtype females only (Fig. S3A). Tryptophan treatment decreased body weight in ss-deficient females only (p < 2.5 × 10^−2^, Fig. S3B). To determine if expression of *ss* affects tryptophan gene expression, we found *vermillion* expression, the *Drosophila* ortholog of TDO, which converts tryptophan to kynurenine, was reduced in ss-deficient flies. We lastly looked at *Cyp6g1*, a cytochrome gene that has previously been shown to be dependent on activation of ss in flies [29]. We found Cyp6g1 expression was unchanged with reduction in ss levels (p < 0.05 for males and females; Fig. S3C–D).

## 4. Discussion

We observed a general decrease in longevity in flies with reduced expression of *ss*, with the *ss* mutation mitigating some of the effects of tryptophan metabolite supplementation. Our findings suggest that *ss*, like the mammalian AhR, has significant impacts on longevity and reproduction and interacts with the tryptophan metabolic pathway. While a hypomorphic mutation in *ss* led to decreased lifespan across both sexes (Fig. 2C–D), we observe a survival advantage early in life among the ss-deficient flies, lasting until approximately 40 to 45 days of age, which is also accompanied by improved stress resistance but decreased fecundity. We confirmed this increase in early life survival by calculating age-specific mortality rates (Fig. 2E–F), and the reduction in early life mortality was more dramatic in females than in males. Collectively, these findings suggest a reproductive trade-off with early-life survival, which is in line with a previous review that characterizes AhR as an antagonistic pleiotropy gene [4]. Our reproductive results are also similar to the deficits observed in AhR mutant worms, which had a reduced number of eggs laid compared to the N2 wildtype [9], and mice, which had a reduced number of pups per litter [10,11]. Our results, while not providing a finite answer to how AhR/ss impacts the aging process across taxa, do increase the current understanding of the role AhR/ss. There is still much to uncover about how AhR influences the aging process, with future research looking further into tissue-specific mechanisms.

Previous work on *ss* mutants has largely focused on insect development [29,30], and more recently has considered the roles of *spineless* in cancer and aging pathways [14,31]. To our knowledge, only one other study has demonstrated the effects of *spineless* on lifespan and health in flies [14]. Cao et al. (2025) found that aged *spineless* mutant flies had markedly reduced lifespans and resilience to oxidative stress and starvation compared to wildtype flies. However, their use of the *Drosophila* line Dmel\ss^1^ (#2973) was constructed from a complete deletion of the *ss*^*1*^ gene from an unspecified wildtype line according to FlyBase. Interestingly, Cao et al. did discover that the gut microbiota-derived tryptophan metabolite indole acetic acid (IAA) may increase lifespan through activation of *ss* in flies [14]. They provide a potential mechanism by which AhR extends lifespan by upregulating Sirt2 through IAA activation. Although their findings are similar to ours, showing significantly reduced lifespans in flies with altered expression of *ss* (Fig. 2C–D), we found that their extremely shortened lifespan makes it hard to determine whether certain AhR-targeted treatments are reactive to ss in flies. Additionally, they also found that aged ss-knockouts had much lower survival rates under oxidative and starvation stressors. Their results run counter to our conclusions regarding survivability under high-stress (oxidative or starvation) conditions; however, this is most likely due to the complete deletion of ss compared to the hypomorph used in this study. Our results indicate that reduction in ss activity may lead to improvement in early and mid-life health while complete ablation has large negative consequences.

In early-aged *ss* hypomorphs, we observed improved resistance to oxidative stress, possibly due to their possessing lower levels of ROS. Prior work found that reduced *ss* expression resulted in reduced ROS concentrations [32], similar to mammalian AhR. This may indicate that at least in early life, ss-deficient animals are protected from outside oxidative stress until later in life. We hypothesized this may be due to reduced *Cyp6g1* expression, a *Drosophila* cytochrome, based on a previous study showing *Cyp6g1* expression induction relies on *ss* [29]. However, we found that cytochrome *Cyp6g1* did not exhibit altered expression in response to changes in *ss* expression (Fig. S3C–D), suggesting that a reduction in oxidative stress through modulation of this cytochrome gene may not be the mode of action for the observed early-life improvements. Therefore, the mechanisms underlying this early-life stress resistance in ss-deficient flies remains unknown. Further interrogation of other *Drosophila* cytochrome genes is necessary to elucidate the mechanism underlying their increased stress resistance.

Another possible explanation for ss flies exhibiting higher stress resilience is their increased body weight; however, we found that only ss females are heavier than wildtype, with no difference in males (Fig. S2B). A larger body mass might contribute to enhanced stress resilience, improved starvation resistance, and poorer climbing ability, which were all more pronounced in female ss-deficient flies. Conversely, Williams et al. measured overall activity in young flies with a null mutation in ss [33], finding that flies with removal of ss actually exhibited increased overall movement [33]; however, this measurement does not necessarily correlate with climbing ability, which is commonly used to measure neuromuscular health. Moreover, AhR−/− mice spent more time on a rotarod and fell off at a higher velocity compared to AhR+/+ mice [33]. However, recent studies are more in line with our findings, showing ss null flies have poorer climbing ability [14]. In addition, studies in worms indicate either decreased motility when ahr-1 is mutated [9] or no differences in motility in ahr-1 mutants [34], but ahr-1 mutant worms may have increased pharyngeal compared to wildtypes [6]. Overall, findings on the effects of AhR on motility are inconsistent across species, and future studies are needed to determine these context dependent effects of AhR on physical function.

We found that direct supplementation with high levels of tryptophan and tryptamine significantly reduced lifespan across sexes and genotypes, but *ss* mutants exhibited significantly less severe consequences from both. In other words, reduced expression of *ss* mitigated some of the effects of tryptophan metabolite supplementation. This suggests that tryptophan metabolites may interact with ss to cause downstream health effects. In line with these findings, we also showed that ss-deficient flies exhibit significantly decreased *vermillion* expression (Fig. S3C–D). *Vermillion* is the *Drosophila* ortholog of TDO in mammals, which converts tryptophan to kynurenine. Previous work has shown that AhR upregulation leads to upregulation of IDO (another TDO ortholog in mammals), which increases kynurenine production, which can positively feedback to upregulate AhR [35]. These results, combined with our gene expression analysis of a *Drosophila* cytochrome, suggest that similar to mammals, the *Drosophila* AhR ortholog interacts with the tryptophan metabolic pathway, but unlike mammals, may not regulate cytochrome genes. Future work will need to include cell-based reporter assays to measure ligand-specific binding of tryptophan and tryptamine to ss in order to determine true causality.

While our study is one of the first to show the effects of *spineless* on longevity and health, it does have several limitations. First, our genetic *spineless* mutant is on a mini-white background, which could potentially affect the expression of tryptophan genes, as tryptophan genes modify eye color in *Drosophila*. However, the gene expression of *vermillion* itself should not be affected. Future studies will need to look at the effects of spineless in other genetic backgrounds for their interactions with tryptophan metabolites. Next, we used only a single high dose of tryptophan and tryptamine and did not explore the underlying mechanisms behind the ss and tryptophan interaction effects. Lastly, the use of a climbing assay to assess neuromuscular function may not be ideal because ss-deficient flies have abnormal leg development. Future studies should examine other facets of health, like cognition. Additionally, future studies should examine the mechanistic changes responsible for the observed early-life benefits and late-life detriments.

## 5. Conclusions

Overall, we observed an early-life survival advantage in *ss* individuals, with increased stress resilience until around 40 days of age. In addition to being more stress resistant, ss-deficient flies were shielded from some of the negative effects of tryptophan and tryptamine supplementation. Our results suggest that similar to mammalian AhR, *Drosophila* ss interacts with the tryptophan metabolic pathway, and *Drosophila* is a viable model for further studies investigating the roles of AhR in aging and health.

## Supplementary Material

1

2

3

4

## Appendix A. Supplementary data

Supplementary data to this article can be found online at https://doi.org/10.1016/j.biochi.2026.02.011.
