# Supplementary material for: The Drosophila aryl hydrocarbon receptor ortholog, spineless, modulates survival and reproduction
Source: Biochimie. Author manuscript; Available in PMC 2026 Sep 4. (PMC13543005; doi:10.1016/j.biochi.2026.02.011)
Supplement: Supplementary file 1 [file NIHMS2207101-supplement-1.pdf]

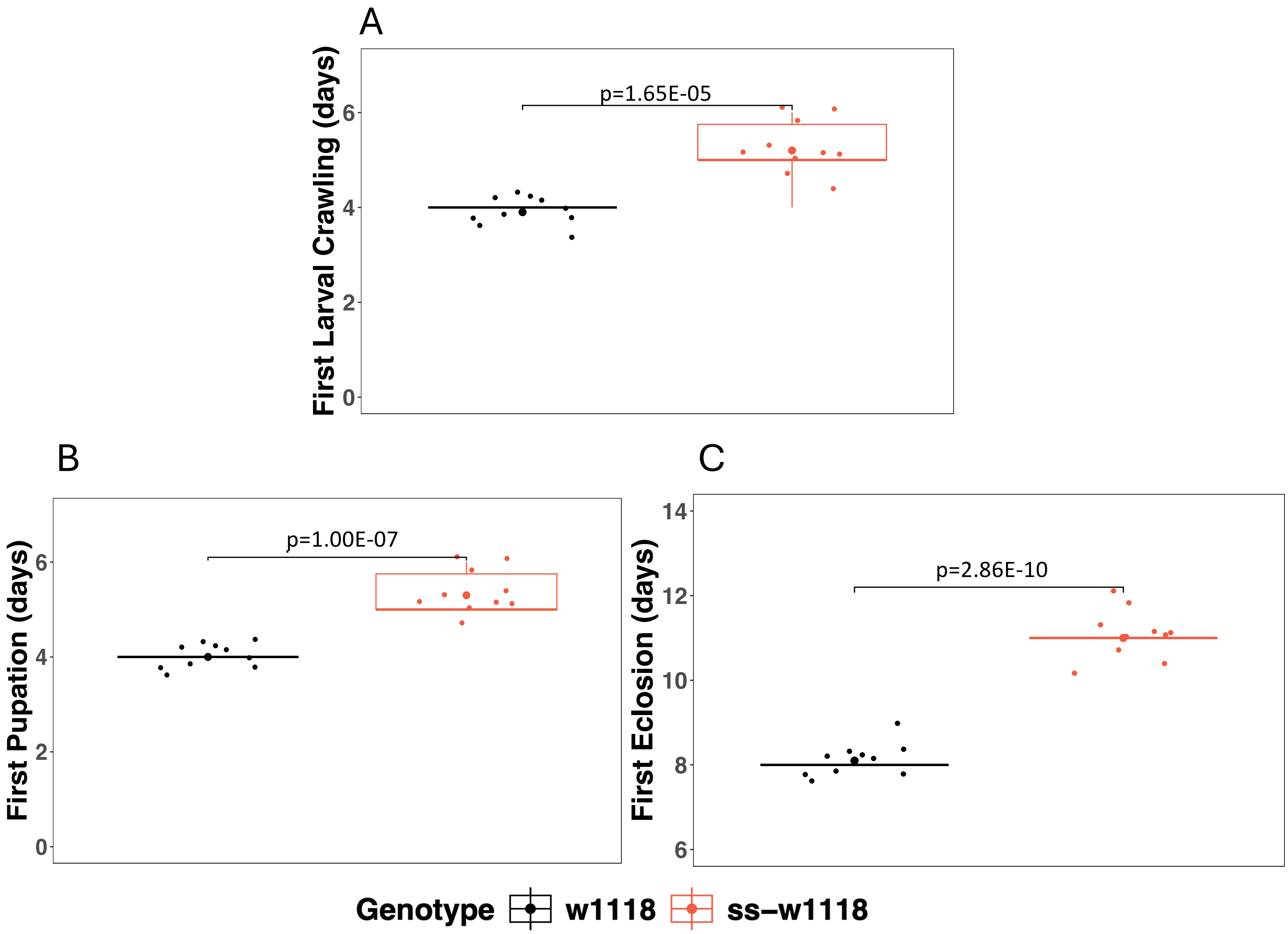
A
First Larval Crawling (days)
p=1.65E-05
B
First Pupation (days)
p=1.00E-07
C
First Eclosion (days)
p=2.86E-10
Genotype
w1118
ss–w1118
