# Supplementary material for: The Drosophila aryl hydrocarbon receptor ortholog, spineless, modulates survival and reproduction
Source: Biochimie. Author manuscript; Available in PMC 2026 Sep 4. (PMC13543005; doi:10.1016/j.biochi.2026.02.011)
Supplement: Supplementary file 2 [file NIHMS2207101-supplement-2.pdf]

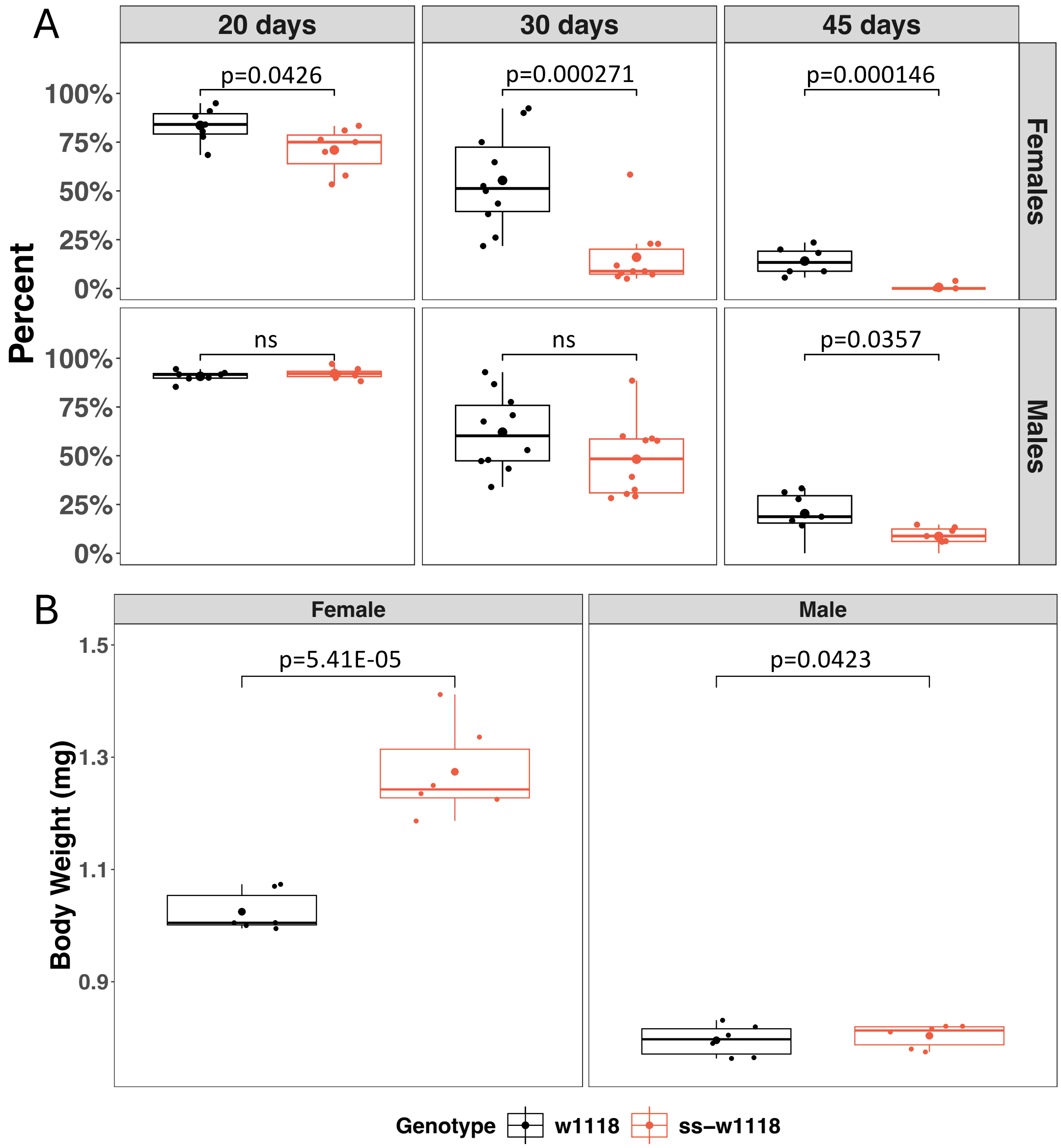
A
20 days
30 days
45 days
Females
Males
Percent
100%
75%
50%
25%
0%
p=0.0426
p=0.000271
p=0.000146
ns
ns
p=0.0357
B
Female
Male
Body Weight (mg)
1.5
1.3
1.1
0.9
p=5.41E-05
p=0.0423
Genotype
w1118
ss–w1118
