# Supplementary material for: The Drosophila aryl hydrocarbon receptor ortholog, spineless, modulates survival and reproduction
Source: Biochimie. Author manuscript; Available in PMC 2026 Sep 4. (PMC13543005; doi:10.1016/j.biochi.2026.02.011)
Supplement: Supplementary file 3 [file NIHMS2207101-supplement-3.pdf]

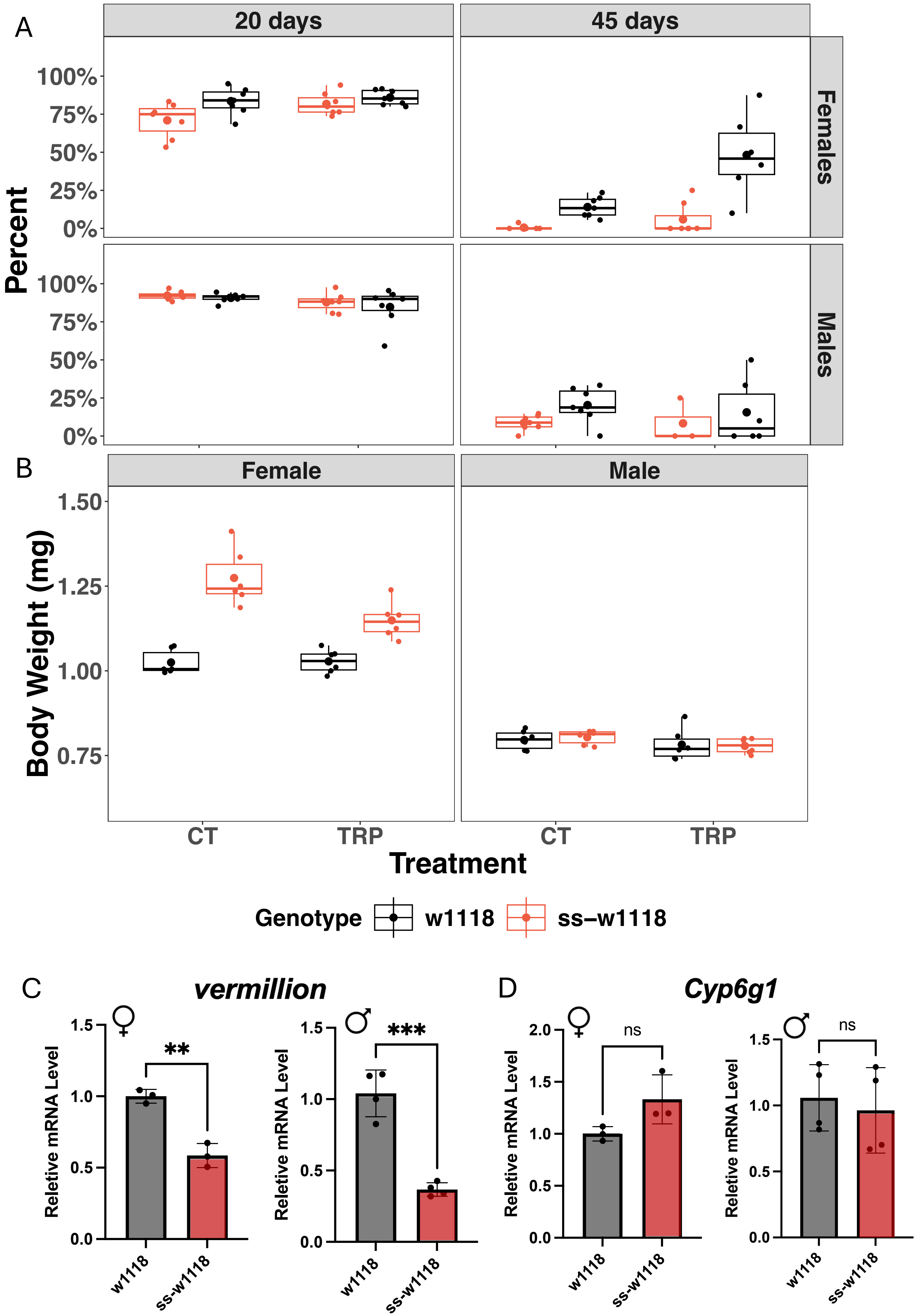

A
20 days
45 days
Females
Males
Percent
100%
75%
50%
25%
0%
B
Female
Male
Body Weight (mg)
1.50
1.25
1.00
0.75
CT
TRP
Treatment
Genotype
w1118
ss–w1118
C
vermillion
Reletive mRNA Level
1.5
1.0
0.5
0.0
♀
♂
**
***
w1118
ss-w1118
D
Cyp6g1
2.0
ns
